# A Comparison of Chest Compression Quality Delivered During On-Scene and Ground Transport Cardiopulmonary Resuscitation

**DOI:** 10.5811/westjem.2016.6.29949

**Published:** 2016-07-19

**Authors:** Christopher S. Russi, Lucas A. Myers, Logan J. Kolb, Christine M. Lohse, Erik P. Hess, Roger D. White

**Affiliations:** * Mayo Clinic, Department of Emergency Medicine, Rochester, Minnesota; † Mayo Clinic, Division of Biomedical Statistics and Informatics, Rochester, Minnesota; ‡ Mayo Clinic, Gold Cross, Rochester, Minnesota; § Mayo Clinic, Division of Health Care Policy and Research, Rochester, Minnesota; ¶ Mayo Clinic, Division of Cardiovascular and Thoracic Anesthesia, Division of Cardiovascular Diseases, Rochester, Minnesota

## Abstract

**Introduction:**

American Heart Association (AHA) guidelines recommend cardiopulmonary resuscitation (CPR) chest compressions 1.5 to 2 inches (3.75–5 cm) deep at 100 to 120 per minute. Recent studies demonstrated that manual CPR by emergency medical services (EMS) personnel is substandard. We hypothesized that transport CPR quality is significantly worse than on-scene CPR quality.

**Methods:**

We analyzed adult patients receiving on-scene and transport chest compressions from nine EMS sites across Minnesota and Wisconsin from May 2008 to July 2010. Two periods were analyzed: before and after visual feedback. CPR data were collected and exported with the Zoll M series monitor and a sternally placed accelerometer measuring chest compression rate and depth. We compared compression data with 2010 AHA guidelines and Zoll RescueNet Code Review software. CPR depth and rate were “above (deep),” “in,” or “below (shallow)” the target range according to AHA guidelines. We paired on-scene and transport data for each patient; paired proportions were compared with the nonparametric Wilcoxon signed rank test.

**Results:**

In the pre-feedback period, we analyzed 105 of 140 paired cases (75.0%); in the post-feedback period, 35 of 140 paired cases (25.0%) were analyzed. The proportion of correct depths during on-scene compressions (median, 41.9%; interquartile range [IQR], 16.1–73.1) was higher compared to the paired transport proportion (median, 8.7%; IQR, 2.7–48.9). Proportions of on-scene median correct rates and transport median correct depths did not improve in the post-feedback period.

**Conclusion:**

Transport chest compressions are significantly worse than on-scene compressions. Implementation of visual real-time feedback did not affect performance.

## INTRODUCTION

The 2010 American Heart Association (AHA)/International Liaison Committee on Resuscitation (ILCOR) Cardiopulmonary Resuscitation (CPR) Guidelines call for a minimum chest compression rate of 100 to 120 compressions per minute and a minimum chest compression depth of 1.5 to 2 inches (3.75–5 cm).^1^ Two clinical studies have reported the quality of chest compressions delivered before emergency medical services (EMS) transport and the quality of those delivered during transport.^2^,^3^ Further evidence has suggested that visual, automated CPR feedback improves CPR quality.^4^,^5^

Research with the use of mannequins has shown that CPR quality is inferior on a moving stretcher^6^ and in a moving ambulance.^7^ We hypothesized that the quality of CPR during ambulance transport is significantly worse than CPR delivered in a static situation (“on scene”—e.g., on the ground or in the street). The aim of this study was to compare the quality of CPR delivered by paramedics on scene to the quality of CPR delivered by paramedics during transport in two distinct periods: before the use of visual feedback (“pre-feedback”) and after the use of visual feedback (“post-feedback”). Visual feedback was deployed systemwide during the study period, and we thought that this was an important confounding variable that could not be ignored; hence, we used two distinct periods with matched cases.

## METHODS

### Study Setting and Population

Mayo Clinic Medical Transport (MCMT) is a nine-site EMS system with a public service area in Minnesota and Wisconsin. In October 2009, MCMT incorporated CPR feedback technology using a sternally positioned accelerometer to quantitatively measure the quality (i.e., the rate and depth) of CPR. We conducted this study with the nine EMS sites in Minnesota and Wisconsin where prehospital care is provided by MCMT’s Gold Cross Ambulance Service. Resuscitation protocols are identical at each site. This is an a priori secondary analysis of data obtained from a large recently published prospective multicenter clinical trial on adult patients treated with CPR in the prehospital environment.^8^ We included adults (age ≥18 years) who had nontraumatic out-of-hospital cardiac arrest, received CPR on scene, and were subsequently transported with ongoing CPR in an ambulance. The null hypothesis was that there is no difference in proportional delivery of correct CPR depth and rate between on-scene CPR and transport CPR.

### Data Collection and Processing

We obtained institutional review board approval from the hospitals that received patients.

Demographic data and transport times were collected with the use of Zoll RescueNet Code Review data and documentation software (Zoll Medical Corp). CPR quality indicators (rate and depth) were abstracted with the use of Zoll M series CPR accelerometer technology and entered into an Excel database (Microsoft Corp). We categorized CPR depth and rate as “above (deep),” “in,” or “below (shallow)” the target range according to AHA guidelines.

### Classification of Time Periods and Quality of CPR

In October 2009, MCMT and Gold Cross Ambulance Service incorporated visual CPR feedback technology (Zoll Medical Corp) in all sites with a sternally positioned accelerometer to quantitatively measure the quality (rate and depth) of CPR. At all times during the study period, prehospital providers were required to use a metronome during CPR, although verification of its use was not possible.

Correct CPR depth was defined as compressions of 1.5 to 2 inches (3.75–5 cm). Correct CPR rate was defined as 100 to 120 compressions per minute. We calculated the proportions of correct CPR depths and rates as percentages of all compressions administered during each CPR episode. Hands-off time was not measured during this study. Only periods when compressions were done were analyzed. We analyzed all data with JMP Version 8.0 statistical software (SAS Institute, Inc).

### Outcomes

The primary outcome was the proportional rate and depth of CPR compressions delivered during on-scene resuscitation and during transport resuscitation. Secondary outcomes were survival to admission and discharge.

### Statistical Analysis

For comparisons of proportional rate and depth of compressions between on-scene and transport resuscitations, we analyzed the cohort in two groups: pre-feedback period (without visual feedback from the Zoll monitor) and post-feedback period (with visual feedback from the Zoll monitor) because we considered this a substantial confounding variable. We used the Wilcoxon signed rank test for paired patient data in each group and the Kruskal-Wallis test for nonpaired data. We used simple descriptive statistics for measures of central tendency to describe demographic and time data. All probability tests were 2-tailed with an α level of .05.

## RESULTS

### Participants

A total of 140 adults had CPR performed on scene and were then transported and required CPR at some time during transport.

### Descriptive Data

Table 1 summarizes cohort demographic features, including age, sex, and on-scene and transport times and distances. There were no significant differences between groups when compared by visual feedback period (Table 2).

### Main Results

In the pre-feedback period, we analyzed 105 of 140 paired cases (75.0%); in the post-feedback period, 35 of 140 paired cases (25.0%) were analyzed (Figure).

#### Pre-feedback Period (n=105)

The proportion of correct depths during on-scene compressions (median, 41.9%; interquartile range [IQR], 16.1%–73.1%) was higher compared to the paired transport proportion (median, 8.7%; IQR, [2.7%–48.9%]). Paired analysis with the Wilcoxon signed rank test showed that the difference was significant (*p*<0.0001). The proportion of correct compression rates during on-scene CPR (median, 45.5%; IQR, [9.9%–60.7%]) was higher compared to the paired transport proportion (median, 11.1%; IQR, [5.8%–34.5%]). Paired analysis with the Wilcoxon signed rank test showed that the difference was significant (*p*<0.0001).

#### Post-feedback Period (n=35)

The proportion of correct depths during on-scene compressions (median, 75.7%; IQR, [36.3%–95.1%]) was higher compared to the paired transport proportion (median, 14.0%; IQR, [4.8%–90.8%]). The difference was significant (*p*<0.0001). The proportion of correct compression rates during on-scene CPR (median, 48.2%; IQR, [14.7%–62.4%]) was higher compared to the paired transport proportion (median, 19.0%; IQR, [9.5%–60.2%]). The difference was not significant (*p*=0.079).

### Other Analyses

#### Effect of Visual Feedback on On-Scene and Transport CPR

Proportions of on-scene median correct rates and transport median correct depths did not improve after the initiation of visual feedback (Kruskal-Wallis test; *p*=0.28 and 0.07, respectively). However, proportions of on-scene median correct depths and transport median correct rates improved significantly (*p*=0.0006 and *p*=0.03, respectively).

#### Effect of Visual Feedback on Mortality

Mortality data were complete for 100 of the 140 patients (71.4%). We categorized mortality as either “discharged alive” or “died as inpatient.” In this subgroup, the Fisher exact test showed no statistically significant difference in survival after implementation of visual feedback (*p*=0.28, odds ratio, 0.48; 95% CI, [0.15–1.58]).

## DISCUSSION

### Summary of Major Findings

Our study showed that, without visual feedback technology, the depth and rate of compressions during CPR while transporting a patient were significantly worse than during on-scene resuscitation. However, both depth and rate were suboptimal, regardless of the environment where the resuscitation occurred.

Despite our expectation that visual feedback technology during CPR would assist in providing the correct rate and depth of compression, regardless of location, we showed that compression depth was statistically worse for patients receiving CPR while being transported in an ambulance compared to patients receiving on-scene CPR. The correct rate during transport compared to on-scene CPR was not significantly different; however, despite the study not having the power to detect a difference, the median absolute percentage difference of 29.2% is concerning. That is, the CPR rate during transport was nearly one-third worse than the rate during on-scene resuscitation despite its lack of statistical significance.

Further, a subgroup analysis was done on the 71.4% of patients for whom full mortality data existed. Visual feedback provided no statistically significant improvement in mortality as measured by discharge from the hospital.

### Strengths and Limitations of the Study

This project had several important limitations. First, we did not account for provider fatigue as a factor in poor CPR performance. Data for this confounder are impossible to collect from transport records. Our suspicion is that on-scene fatigue was not a factor because our practice is to change providers every two minutes, but it could confound the transport phase results because often only one provider is in the patient compartment. However, this effect is likely small given that the median transport time was six minutes.

We did not account for the provider type and aerobic health of the provider. Our providers, as is likely true in most systems, include EMTs, paramedics, and firefighters who have a broad range of physical abilities.

Our study did not account or measure hands-off time as has been done in previous studies. Our analysis quantified only the periods when compressions were done in each respective resuscitation phase (on scene vs transport). Hands-off time has been clearly associated with increased mortality; however, our aim was to show the quality of what was delivered rather than the amount.

To our knowledge, the Zoll accelerometer has not been prospectively validated with outcomes assessment (i.e., survival) in moving ambulances.

### Comparison with Other Published Studies

To our knowledge, this study is the third showing how transport affects the quality of CPR delivered. Olasveengen et al^2^ demonstrated similar findings with a focus on the rate and the compression ratio. A recent study found that patients achieving out-of-hospital return of spontaneous circulation (ROSC) experienced another cardiac arrest 38% of the time.^9^ These high rates of rearrest in patients with ROSC show the need to not only anticipate the high potential for rearrest in those transported but the need to improve CPR quality during transport.

Inappropriate compression depth has been associated with worse outcomes.^5^,^10^ In the present study, the difference between the proportion of correct depths during on-scene compressions (median, 75.7%) and the proportion of correct depths during transport (median, 14.0%) was 61.7%.

Debate about CPR devices and marketing of mechanical CPR devices has grown along with evidence of their potential efficacy.^11^,^12^ The key element in the present study is that the on-scene environment is a relatively static environment in which to perform CPR compared to the more dynamic environment of the patient compartment in an ambulance. Although on-scene CPR may occur in challenging and austere locations, the on-scene environment does not include the types of external acceleration forces experienced in a moving ambulance.

An additional factor likely contributing to poor CPR performance in an ambulance is the change in rescuer posture. In the on-scene environment, the patient is often on the floor or on the ground, so that the rescuer can kneel beside the patient. In an ambulance, the rescuer must stand and lean because the patient is on a cot, fairly low to the floor. Studies have determined the ideal bed height for optimal compression quality and have shown that a rescuer’s performance is worse in a standing position than in a kneeling.^13^ However, secured ambulance cots are not adjustable.

We believe that future research needs to address how to create a more static environment for performing quality CPR during transport. Most patients in the present study had ROSC before transport and were presumed to have a much higher likelihood of survival. However, a concerted effort should always be made to stabilize a patient’s condition before transport, but if rearrest occurs during transport, CPR must be resumed in the ambulance. If quality CPR cannot be provided during transport arrest, several questions need to be considered:

1. Should protocols mandate that EMS providers stop the ambulance if CPR resumption is necessary?
2. Should EMS providers have mechanical CPR devices at their disposal in anticipation of the need for CPR during transport?
3. Should the monitors be placed in an optimal visual position in the ambulance to take full advantage of the visual feedback markers?
4. Should clear audio feedback be provided at all times, augmenting the visual feedback?
5. Should extra crew members be in the patient compartment of the ambulance to serve as coaches?

Our study showed several interesting results. Clearly, the transport period, regardless of visual feedback, had a significantly worse quality of CPR rate and depth during compressions. On-scene administration of compressions of the correct depth improved significantly after implementing visual feedback; however, the other quality markers showed no improvement or marginal benefit.

## CONCLUSION

Despite the presence or absence of a visual feedback tool during cardiopulmonary arrest, in a comparison with on-scene CPR, the quality of CPR delivered during transport was significantly worse. Further work should assess the effects of ambulance monitor locations, ambulance configurations to improve rescuer position, audio feedback systems or coaches, and mechanical CPR devices.

## Figures and Tables

**Figure:**
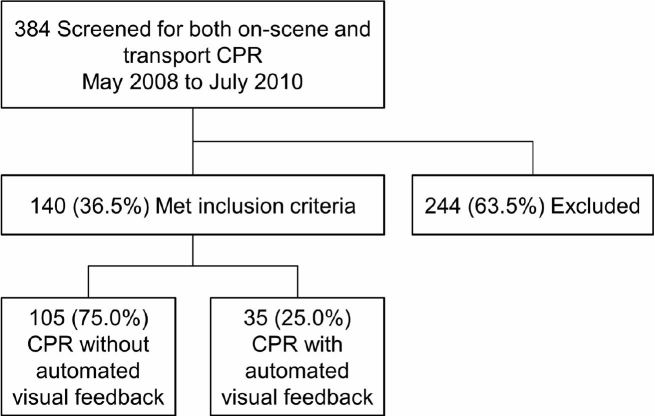
Patient flowchart. Cardiopulmonary resuscitation (CPR) was administered without automated visual feedback to 105 patients; CPR was administered with automated visual feedback to 35 patients.

**Table 1:** Cohort demographic and clinical features.

Feature	Value
Age, mean (95% CI), y	65.6 (62.9–68.2)
Male, % of patients (95% CI)	67.4 (58.9–74.9)
First rhythm, median (%)^a^	
Ventricular fibrillation	39 (30.2)
Ventricular tachycardia	1 (0.8)
Nonshockable (pulseless electrical activity and asystole)	86 (66.7)
Not documented	3 (2.3)
On-scene care time, median (IQR), min	18.7 (11.2)
Estimated patient weight, mean (95% CI), kg	91.2 (86.3–96.1)
Distance from scene to hospital, median (IQR), km	5 (10)
CPR duration, median (IQR), min	
On-scene	7.7 (10.1)
Transport	2.2 (3.3)
Correct rate, median (IQR), %	
On-scene	45.8 (49.8)
Transport	11.5 (39.3)
Correct depth, median (IQR), %	
On-scene	48.3 (61.0)
Transport	9.8 (54.8)
Disposition, No. of patients (%)	
Died as inpatient	67 (51.9)
Discharged alive	33 (25.6)
Hospitalized at end of study	29 (22.5)

*CPR*, cardiopulmonary resuscitation; *IQR,* interquartile range; *CI,* confidence interval

a Documented by Gold Cross Ambulance personnel

**Table 2:** Cohort descriptive features by visual feedback period.

Feature	Pre-feedback (n=105)	Post-feedback (n=35)	*P*^a^
Age, median (IQR), y	66.0 (22.0)	66.5 (23.5)	.86
Male, %	67.4	67.6	.98
Time, median (IQR), min			
On-scene	19.2 (10.4)	18.3 (12.8)	.62
Transport	6.0 (5.0)	6.0 (6.1)	.78
Destination, median (IQR), km			
To scene	5.0 (6.7)	5.0 (8.3)	.40
To hospital	5.0 (10.0)	4.0 (11.7)	.21

*IQR*, interquartile range

a Comparison of medians with use of nonparametric tests (Wilcoxon signed rank test)
